# Sequential IVM by CNP preincubation and cooperating of PGE2 with AREG enhances developmental competence of SCNT reconstructs in goat

**DOI:** 10.1038/s41598-022-08238-5

**Published:** 2022-03-10

**Authors:** Nazanin Assareh, Mahya Shahemabadi, Shiva Rouhollahi Varnosfaderani, Farnoosh Jafarpour, Mehdi Hajian, Mohammad Hossein Nasr-Esfahani

**Affiliations:** 1 Department of Biology, Faculty of Science and Technology, ACECR Institute of Higher Education (Isfahan), Isfahan, Iran; 2 Department of Animal Biotechnology, Reproductive Biomedicine Research Center, Royan Institute for Biotechnology, ACECR, Isfahan, Iran

**Keywords:** Biotechnology, Developmental biology

## Abstract

Developmental competence of in vitro matured cumulus oocyte complexes (COCs) in conventional IVM (C.IVM) is lower than in vivo maturated COCs and is related to unsynchronized nuclear and cytoplasmic maturation. To overcome this dearth, COCs can be exposed to granulosa secreted factors in a two-step system. Therefore, in the first experiment, 1000 nM of C-type natriuretic peptide for 8 h was determined (CAPA), as the best time and concentration to retain oocytes in germinal vesicle stage. This condition, also reduces lipid droplets and increases the expression of *ATGL* and *PLIN2* involved in lipolysis and lipogenesis, respectively. In the second experiment, maturation was stimulated with prostaglandin E2 and amphiregulin for 18 h (CAPA-IVM), and their optimal concentrations based on blastocyst formation rates through in vitro fertilization (IVF) were determined as 1 and 600 nM, respectively. In the third experiment, the in vitro and in vivo developmental competency of SCNT embryos in CAPA-IVM group were determined. Despite similar blastocyst formation rates in IVF and SCNT between CAPA-IVM and C.IVM, the quality of blastocysts were quality was higher in CAPA-IVM, which reflected itself, as higher ICM/TE ratio and also expression of *NANOG* in SCNT blastocysts. Pregnancy rate, live births rate and SCNT efficiency were not significant between CAPA-IVM and C.IVM groups. Therefore, CAPA-IVM can improve the developmental competency of SCNT derived embryos.

## Introduction

The first report of pregnancy following in vitro maturation (IVM) was published in human in 1991 by Cha KY et al.^1^. Despite the successful efficiency of IVM in assisted reproductive technology (ART), its efficiency is limited as compared with in vivo maturation^2^. The microenvironment surrounding the immature oocyte includes follicular fluid, theca cells, and granulosa cells (GCs), each of which have a vital and determinant role in meiotic progression, cytoplasmic maturation, and cumulus expansion^3,4^. Previous studies demonstrated that biomarkers related to GCs play a pivotal role in successful embryo development and pregnancy outcomes^5^.

Previous researches have shown that co-culture of COCs with GCs improves the maturation efficiency and developmental competence of oocytes in various mammalian species including mice^6^, cattle^7^, and human^5^. Granulosa and cumulus cells produce epidermal growth factor (EGF)-like proteins [including amphiregulin (AREG), epiregulin (EREG) and betacellulin (BTC)], prostaglandins (PGs), natriuretic peptides (NPs), brain-derived neurotrophic factor (BDNF), and insulin-like growth factors (IGF) that regulate the maturation and metabolism of COCs^6,8–10^. Following the luteinizing hormone (LH) surge, EGF-like proteins act on EGF-receptor and induce the expression of extracellular signal-regulated protein kinase 1/2 (ERK1/2) in granulosa and cumulus cells (CCs), which consequently leads to the expression of genes related to cumulus expansion^11,12^. One of the important target genes of the ERK1/2 pathway is prostaglandin synthase 2 (PTGS2), which leads to the production of prostaglandin E2 (PGE2) through a positive feedback loop between AREG and PGE2. PGE2 is considered as the main PGs secreted following the induction of PTGS2 by maturing COCs^13^. Recent studies in various species such as human^14^, rhesus monkey^15^, and diverse domestic animals^16,17^ demonstrate the important role of PGE2 during early embryogenesis.

C-type natriuretic peptide (CNP) is the major NPs and binds to natriuretic peptide receptor 2 (*NPR2*) that is mainly present in CCs. Apparently, the bovine is an exception and in addition to CCs, denude oocytes (DOs) also have *NPR2*^18^. Induction of CNP markedly leads to the synthesis of cyclic guanosine monophosphate (cGMP) in CCs which is transferred to the oocyte via gap junctions.

Cyclic GMP has suppressive effect on phosphodiesterase 3A (PDE3A), which maintains the concentration of cyclic adenosine monophosphate (cAMP) at high level in the oocytes and sustains them in germinal vesicle (GV) stage^6^. Recent studies have demonstrated the influence of CNP on meiosis resumption in swine^19^, bovine^20^, ovine^10^ and other species^6,21^.

Some studies have shown that manipulating the level of cGMP with CNP in the maturation medium decreases the lipid content in the oocyte^22^. Lipolysis results from the sequential activity of adipose triglyceride lipase (ATGL), hormone-sensitive lipase (HSL), and mono-acyl glycerol lipase (MAG)^23^. ATGL, as a member of a family of lipid metabolizing enzymes in mammals, is primarily located on the surface of the lipid droplet. Activation of ATGL plays key role in initiation of lipolysis^24^. Also, perilipin (PLIN2) and diacylglycerol acyltransferase 1 (DGAT1) are required for the accumulation and stability of the LDs^22^. Higher cytoplasmic lipid content observed in in vitro produced (IVP) embryos in compare to their in vivo counterparts is one of the factors responsible for their lower cryosurvival rate^25,26^. Lower lipolysis rate is one of the shortcomings of in vitro matured oocytes as compare with in vivo matured ones, which can be remedied with some metabolites such as forskolin, L-carnitine, and CNP^22,27^.

Two-stage IVM system is designed to “improve the developmental capacity” of in vitro matured oocytes and is therefore called “Capacitation IVM”^28^. This is a pre-maturation culture period intended to maintain oocytes in GV for longer periods, so they would have extra time to prepare for final maturation, and is termed as capacitation period (therefore, CAPA). Subsequently, COCs are exposed to a second culture media for a further period, when meiosis resumption and progression is stimulated (IVM), so the two-step procedure is called Capacitation IVM or CAPA-IVM. In the present study, LH and follicle-stimulating hormone (FSH), two main conventional factors for inducing meiotic progression, were removed from IVM medium and instead, downstream factors including CNP, PGE2 and AREG were used for a two-step maturation system. The effect of COCs exposure to granulosa secreted factors in a two-step system. In this study, the effect of COCs exposure to granulosa secreted factors in a two-step system has been investigated by three consecutive experiments. The first experiment was done to determine best time and concentration of CNP to retain oocytes in GV stage and reduced lipid content. In the second experiment, stimulation of maturation of COCs with PGE2 and AREG for 18 h was carried out and their optimal concentrations based on in vitro blastocyst formation rates were determined. In the third experiment, the in vitro and in vivo developmental capacity of somatic cell nuclear transfer (SCNT) embryos derived by CAPA-IVM were investigated.

## Results

### Experiment 1: the effect of CNP on meiotic arrest, lipid content, and expression of genes related to the lipolysis and lipogenesis

The first item was done to determine best time and concentration of CNP to retain oocytes in GV stage. In 1000 nM CNP for 8 h, percentage (68.8 ± 7.8%) of COCs remaining at the GV stage was higher than other groups (Fig. 2A) and was significantly different from tissue culture medium (TCM) and C.IVM groups (Fig. 2B). While exposing DOs to 1000 nM of CNP for 8 h did not show any changes in the percentage of DOs arrested at the GV stage compared to TCM group (*P* > 0.05, Fig. 2C).

**Figure 1:**
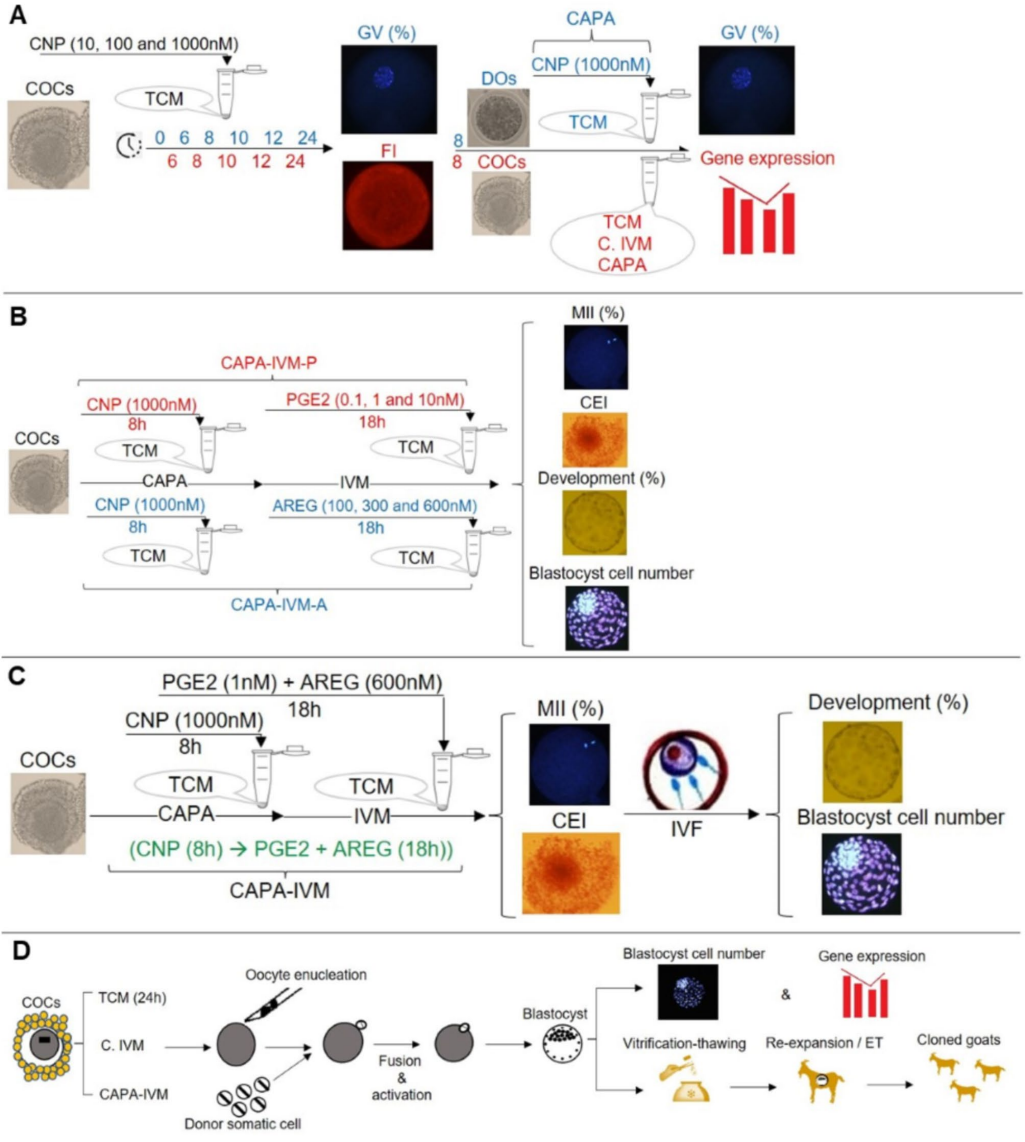
Experimental design. (**A**) COCs were exposed to different concentrations of CNP for different durations. Then, percentage COCs remaining in GV stage, as well as FI for lipid content were determined. 1000 nM CNP for 8 h was selected as the optimal condition and termed CAPA. In addition, DOs were also exposed to CAPA and GV percentage were compared to DOs cultured in TCM. FI and relative gene expression of COCs were also compared between COCs cultured in TCM, C.IVM and CAPA for 8 h. (**B**) COCs were exposed to CAPA, they were exposed to different concentrations of PGE2 and AREG for 18 h. Control group were termed CAPA-TCM. Then, percentage MII, CEI and developmental rate as well as blastocyst cell number were compared with CAPA-TCM. The optimal concentrations for PGE2 and AREG were termed CAPA-IVM-P and CAPA-IVM-A. (**C**) After determined best condition of CNP, PGE2 and AREG, COCs were exposed to CAPA and then exposed to optimal concentrations of PGE2 and AREG (CAPA-IVM) and percentage of MII, CEI and developmental rate as well as blastocyst cell number were compared to TCM (24 h), CAPA-TCM, C. IVM, CAPA-IVM-P and CAPA-IVM-A through IVF. (**D**) COCs were matured in TCM (24 h), C.IVM and CAPA-IVM and subsequently they were used for SCNT procedure. SCNT derived blastocytes from each group were assessed and compared for developmental rates, blastocyst cell number, ICM/TE ratio, gene expression and ability to re-expand after vitrification. Vitrified embryos form C. IVM and CAPA-IVM were transferred to recipient to determine their developmental competency. Cumulus oocyte complexes (COCs); C type natriuretic peptide (CNP); Germinal vesicle (GV); Fluorescent intensity (FI); Capacitation (CAPA); Denuded oocytes (DOs); Tissue culture medium (TCM); Conventional IVM (C.IVM); Prostaglandin E2 (PGE2); Amphiregulin (AREG); In vitro maturation (IVM); Capacitation-TCM (CAPA-TCM); Metaphase II (MII); Cumulus expansion index (CEI); Capacitation IVM-prostaglandin E2 (CAPA-IVM-P); Capacitation IVM-amphiregulin (CAPA-IVM-A); In vitro fertilization (IVF); Somatic cell nuclear transfer (SCNT).

**Figure 2:**
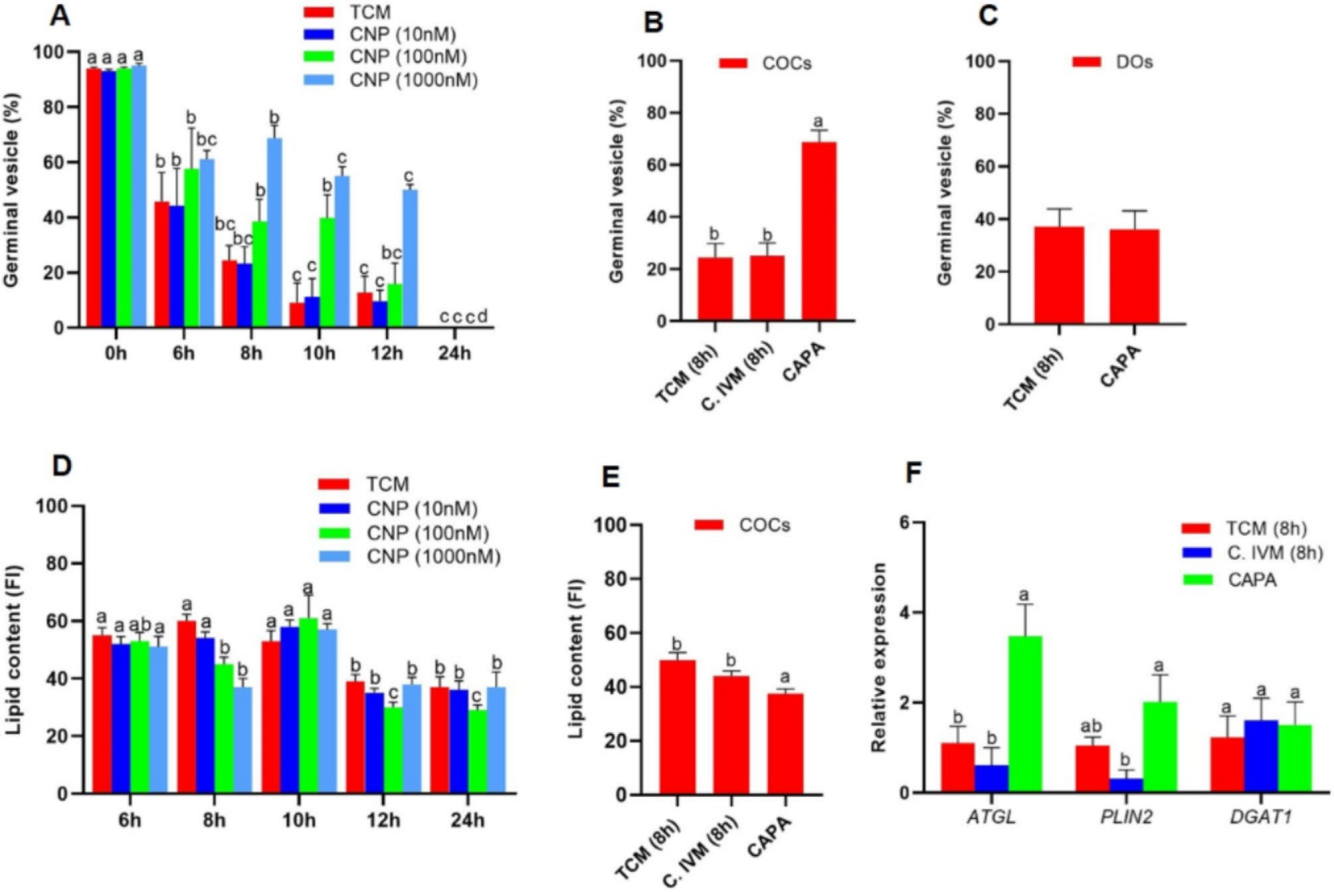
Effect of CNP on meiotic arrest, lipid content, the lipolysis and lipogenesis genes expression. COCs were exposed to different concentration of CNP to determine the optimal concentration and duration for percentage of COCs remaining at the GV stage (**A**), This condition was termed CAPA. Percentage COCs remining in GV stage were also compared between TCM (8 h), C.IVM (8 h) and CAPA (**B**). DOs were exposed to TCM (8 h) and CAPA and percentage DOs remaining in the GV stage were compared (**C**). COCs were exposed to different concentration of CNP, for different duration to determining the FI for lipid content (**D**). Lipid content (**E**) as well as relative genes expression (**F**) were also compared between TCM (8 h), C.IVM (8 h) and CAPA. Cumulus oocyte complexes (COCs); C type natriuretic peptide (CNP); Germinal vesicle (GV); Denuded oocytes (DOs); Tissue culture medium (TCM); Fluorescent intensity (FI); Conventional IVM (C.IVM). (**A**, **B**, **D**–**F**) Different letters are significantly different at *P* < 0.05. (**C**) No significantly difference was observed between groups at *P* > 0.05.

In the next item, we evaluated the effect of CNP on the lipid content of oocytes treated with various concentrations of CNP (Fig. 1A); CNP treatment at both 100 nM and 1000 nM for 8 h significantly reduced the lipid content as compared to TCM group (*P* < 0.05, Fig. 2D). However, at 10 h time point, we observed a significant raise in lipid content of oocytes treated with 100 and 1000 nM CNP compared to 8 h. In addition, the lipid content of oocytes decreased across all groups at 12 and 24 h as compared to previous time points (6, 8 and 10 h) (Fig. 2D). Our data revealed that the lipid content of CNP treated COCs was significantly lower as compared to TCM and C.IVM groups (*P* < 0.05, Fig. 2E). 1000 nM CNP for 8 h was determined as the optimal condition and termed CAPA.

Also, analysis of mRNA expression in COCs treated with CAPA as compared to TCM and C.IVM groups at the same time point showed a significant increase in the relative mRNA abundance of *ATGL*. The relative mRNA abundance of *PLIN2* was significantly higher in CAPA group as compared with C.IVM. The relative mRNA expression of *DGAT1* was similar among all experimental groups (Fig. 2F). Similar results were obtained when *GAPDH* was also instead of *β-actin* as reference gene (data not shown).

### Experiment 2: developmental competence of COCs exposed to CNP, PGE2 and AREG

Here, we aimed to determine the best concentrations of PGE2 and AREG for stimulating maturation for 18 h. In order to determine the developmental capacity of matured COCs in various treatment groups, they were subjected to IVF procedure.

At first, immature COCs were exposed to CAPA after that, they were exposed to different concentrations of PGE2 (0.1, 1, and 10 nM) for 18 h (Fig. 1B). Treatment of immature COCs with 1 nM PGE2 for 18 h showed no effects on cumulus expansion index (CEI) but acquired the highest metaphase II (MII) (44.85 ± 3.41), and blastocyst rates, but no effects on blastocyst cells number compared with the CAPA-TCM (Table 1). The best concentration group (CNP (8 h) → PGE2 (1 nM)) was named CAPA-IVM-P.

**Table 1:** Determination of optimal concentration of PGE2 and AREG in COCs.

Groups	CEI	MII (%)	Cleavage (%)	Blastocyst (%)	No. ICM cell	No. TE cell	No. Total cell
CAPA-TCM	1	26^b^ ± 6.99	83 ± 5.51	20.01^b^ ± 3.95	25 ± 3.46	67.18 ± 7.13	91.90 ± 10.24
CAPA-IVM-P (0.1 nM)	1.30	26.66^b^ ± 4.40	83.49 ± 5.52	33.22^ab^ ± 5.01	23.64 ± 2.24	71.85 ± 6.99	95.50 ± 8.83
CAPA-IVM-P (1 nM)	1.60	44.85^a^ ± 3.41	94.09 ± 4.92	45.53^a^ ± 7.80	26.00 ± 3.86	69.85 ± 8.68	94.42 ± 11.06
CAPA-IVM-P (10 nM)	1.33	26.40^b^ ± 4.75	90.86 ± 8.37	29.59^ab^ ± 7.52	20.57 ± 2.58	66.64 ± 6.27	87.21 ± 7.82
CAPA-TCM	1	12^b^ ± 1.09	83 ± 5.51	19.17^b^ ± 2.92	28 ± 8.55	60.12^b^ ± 8.47	87.87^ab^ ± 15.88
CAPA-IVM-A (100 nM)	1.02	24.26^ab^ ± 0.74	81.87 ± 7.61	27.79^ab^ ± 4.81	28.00 ± 3.77	71.94^ab^ ± 7.43	99.94^ab^ ± 10.31
CAPA-IVM-A (300 nM)	1.47	31.35^ab^ ± 0.15	77.84 ± 7.73	28.50^ab^ ± 4.51	19.81 ± 2.91	62.36^b^ ± 7.30	82.18^b^ ± 9.35
CAPA-IVM-A (600 nM)	1.27	51.80^a^ ± 10.70	95.20 ± 1.7	40.77^a^ ± 4.18	30.00 ± 3.24	99.33^a^ ± 7.62	120.66^a^ ± 12.91

Prostaglandin E2 (PGE2); Amphiregulin (AREG); Cumulus oocyte complexes (COCs); Metaphase II (MII); Cumulus expansion Index (CEI); Capacitation TCM (CAPA-TCM); Capacitation IVM-prostaglandin E2 (CAPA-IVM-P); Capacitation IVM-amphiregulin (CAPA-IVM-A); Inner cell mass (ICM) and Trophectoderm (TE). Different letters are significantly different at *P* < 0.05.

Similar to the previous experiment for PGE2, COCs were exposed to different concentrations of AREG (100, 300, and 600 nM) for 18 h (Fig. 1B). 600 nM of AREG significantly increased the MII rate (51.80 ± 10.70), blastocyst rate and trophectoderm (TE) cell number as compared to the CAPA-TCM (*P* < 0.05, Table 1). No difference was observed in CEI, cleavage rate, inner cell mass (ICM) and total cell number (TCN) among the experimental groups (*P* > 0.05, Table 1). The best concentration group (CNP (8 h) → AREG (600 nM)) was named CAPA-IVM-A. Control group here was CAPA-TCM. In this group, immature COCs were exposed to CAPA and then they were exposed to TCM.

**Figure 3:**
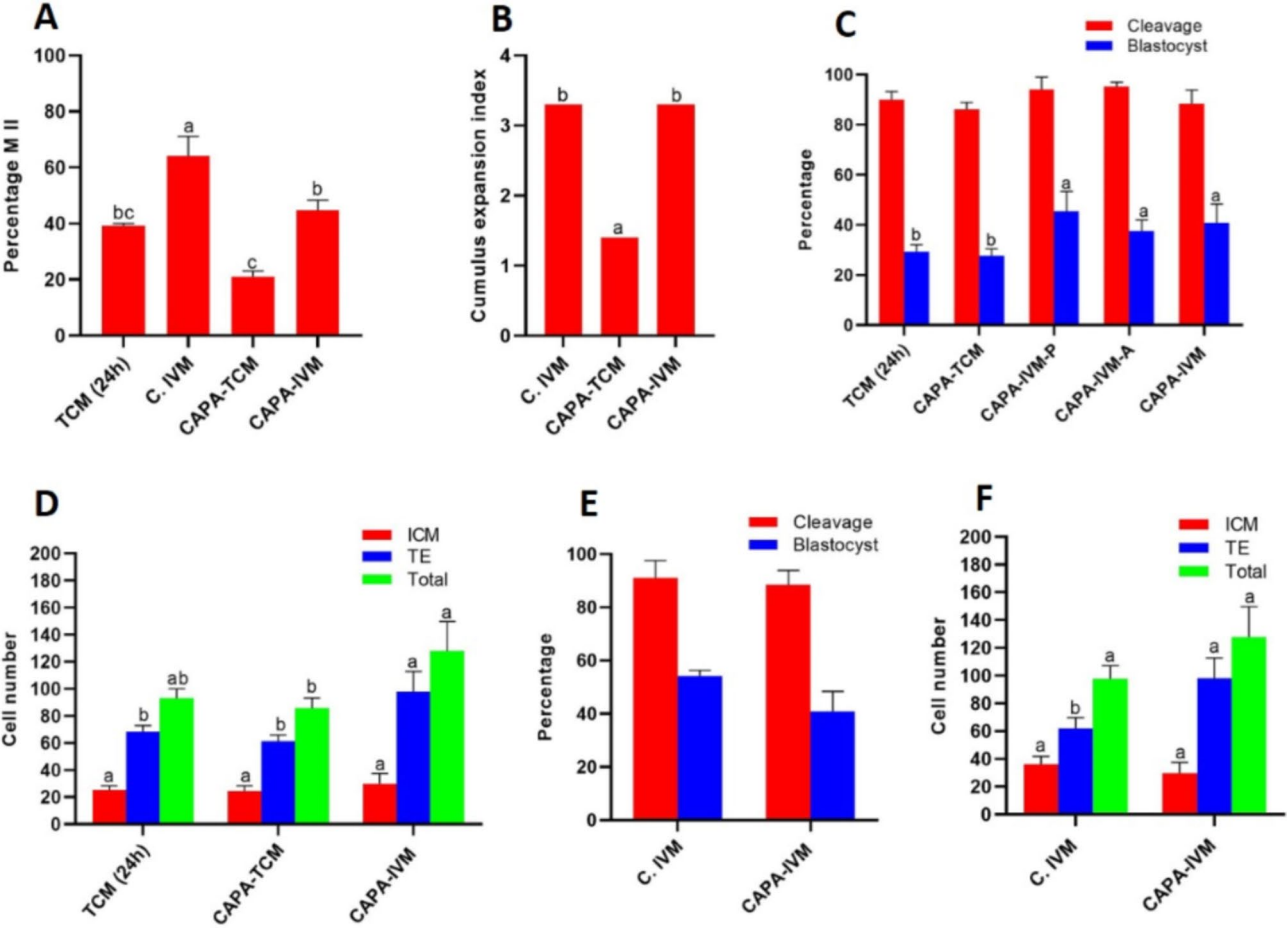
Developmental competence of COCs exposed to CNP, PGE2 and AREG via IVF. COCs were exposed to TCM (24 h), C.IVM, CAPA-TCM, CAPA-IVM-P, CAPA-IVM-A and CAPA-IVM. COCs were exposed to 1000 nM of CNP for 8 h (CAPA) and then were transferred to TCM containing 1 nM PGE2 and 600 nM AREG for 18 h (CAPA-IVM). At this time percentage oocyte in MII (**A**) with their CEI (**B**) were determined. COCs were also used for IVF and their cleavage as well as their blastocyst rate (**E**) were compared between groups (**C**). The quality of blastocyst based in ICM, TE number and TCN were also determined and compared (**D**, **F**). Cumulus oocyte complexes (COCs); Tissue culture medium (TCM); Capacitation TCM (CAPA-TCM); Conventional IVM (C.IVM); Capacitation IVM-prostaglandin E2 (CAPA-IVM-P); Capacitation IVM-amphiregulin (CAPA-IVM-A); Capacitation IVM (CAPA-IVM); C type natriuretic peptide (CNP); Prostaglandin E2 (PGE2); Amphiregulin (AREG); Metaphase II (MII); Cumulus expansion Index (CEI); in vitro fertilization (IVF); Inner cell mass (ICM); Trophectoderm (TE) and Total cell number (TCN). (**A**–**D**, **F**) different letters are significantly different at *P* < 0.05. (**E**) No significantly difference was observed between groups at *P* > 0.05.

In the next item, after determining the best concentration and time of granulosa secreted factors (CNP, PGE2 and AREG) then, this combined treatments (CNP (8 h) → PGE2 (1 nM) + AREG (600 nM) (18 h)) was named CAPA-IVM (Fig. 1C). MII rate was significantly reduced in the CAPA-IVM as compared to the C.IVM (44.66 ± 3.66, 64.28 ± 7.14, respectively) (*P* < 0.05, Fig. 3A). No difference was observed in CEI between the C.IVM and CAPA-IVM groups (*P* > 0.05, Fig. 3B), but it was significantly higher in these groups (C.IVM and CAPA-IVM groups) as compared to CAPA-TCM group (*P* < 0.05, Fig. 3B).

The blastocyst rate was significantly higher in CAPA-IVM-P, CAPA-IVM-A and CAPA-IVM groups as compared to TCM (24 h) and CAPA-TCM groups (*P* < 0.05, Fig. 3C). The quality of IVF-derived blastocysts in terms of the number of TE cells was enhanced significantly in CAPA-IVM group as compared with the TCM (24 h), CAPA-TCM and C.IVM groups (*P* < 0.05, Fig. 3D,F). Our data did not reveal any significant difference between the C.IVM and CAPA-IVM groups in cleavage and blastocyst rates (40.90 ± 7.49 and 54.19 ± 2.09, respectively) after IVF (*P* > 0.05, Fig. 3E).

### Experiment 3: In vitro and in vivo development of SCNT embryos in CAPA-IVM

**Figure 4:**
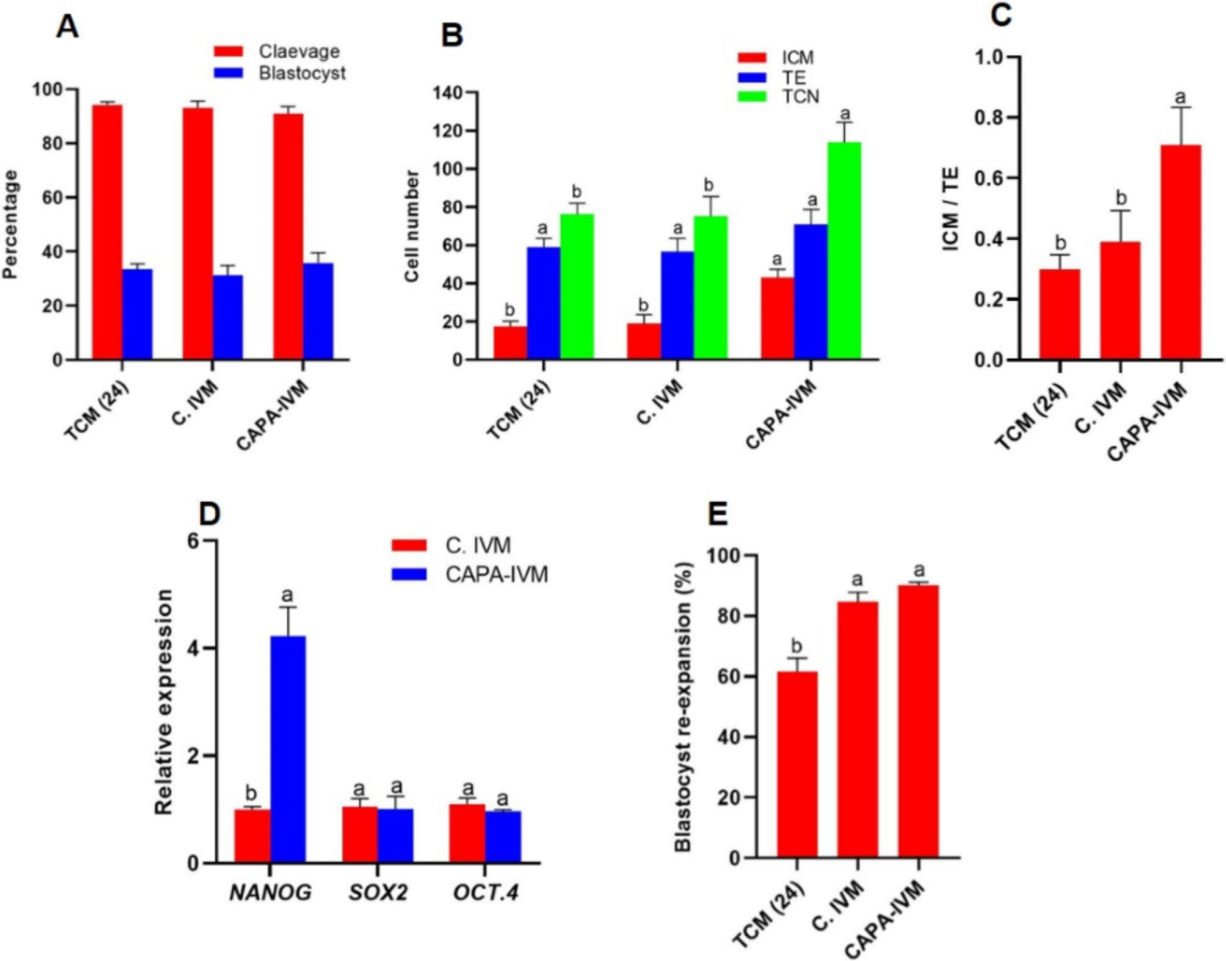
In vitro development of SCNT embryos in CAPA-IVM group. COCs were exposed to TCM (24 h), C.IVM and CAPA-IVM. COCs were exposed to 1000 nM of CNP for 8 h (CAPA) and then were transferred to TCM containing 1 nM PGE2 and 600 nM AREG for 18 h (CAPA-IVM). At this time the oocytes were denuded, enucleated, fused and cultured in SOF for 7 days. Finally, cleavage and blastocyst rates were determined on day 3 and 7, respectively (**A**). The quality of blastocysts was determined based on their ICM, TE number and TCN, as well as their ICM/TE ratio (**B**, **C**). The blastocysts were assessed for the relative expression of pluripotency related genes as well as their quality (**D**). The blastocysts were vitrified and thawed and their re-expansion rate was determined (**E**). Cumulus oocyte complexes (COCs); Tissue culture medium (TCM); Conventional IVM (C.IVM); Capacitation IVM (CAPA-IVM); C type natriuretic peptide (CNP); Prostaglandin E2 (PGE2); Amphiregulin (AREG); Somatic cell nuclear transfer (SCNT); Synthetic oviducal fluid (SOF); Inner cell mass (ICM); Trophectoderm (TE) and Total cell number (TCN). (**B**–**E**) Different letters are significantly different at *P* < 0.05. (**A**) No significantly difference was observed between groups at *P* > 0.05.

In SCNT procedure (Fig. 1D), the cleavage and blastocyst rate (35.75 ± 3.80, 31.18 ± 3.58, respectively) did not show a significant difference among CAPA-IVM and C.IVM groups (*P* > 0.05, Fig. 4A), while the quality of derived blastocysts in terms of ICM, TCN and ICM/TE ratio increased significantly in the CAPA-IVM group as compared with the C.IVM and TCM (24 h) groups (*P* < 0.05, Fig. 4B,C).

Also, to further investigate the developmental competence of the derived blastocysts, the transcription levels of three pluripotency genes (*OCT4*, *NANOG*, and *SOX2*) were determined using quantitative PCR. As shown in (Fig. 4D), remarkably, the relative expression level of *NANOG* was higher in the CAPA-IVM group as compare to C.IVM (*P* < 0.05). The transcription levels of *SOX2* and *OCT.4* were similar between the experimental groups (*P* > 0.05, Fig. 4D). Meanwhile, after thawing of vitrified blastocysts, the percentage of re-expansion rate was evaluated among the experimental groups. The CAPA-IVM group had the highest percentage of re-expansion rate, which was only significant with TCM (24 h) group (*P* < 0.05, Fig. 4E). Finally, as depicted in (Table 2), established pregnancy, full-term pregnancy, loss of pregnancy, live births and SCNT efficiency were not significantly different between CAPA-IVM and C.IVM groups (*P* > 0.05).

**Table 2:** Results of in vivo developmental competence of SCNT goat embryos developed in C.IVM and CAPA-IVM.

Groups	Transferred Embryo	Recipient	Established pregnancy%	Loss of pregnancy%	Full term pregnancy%	Live birth	SCNT efficiency%
C. IVM	30	15	7 (46.66%)	3 (42.85%)	4/7 (57.14%)	4	4/30 (13.33%)
CAPA-IVM	30	15	8 (53.33%)	3 (37.50%)	5/8 (62.50%)	6	6/30 (20%)

*SCNT* Somatic cell nuclear transfer, *C.IVM* Conventional IVM, *CAPA-IVM* Capacitation IVM.

## Discussion

Presence of granulosa secreted factors play an essential role in oocyte in vitro maturation^29,30^. Oocyte quality is one of the main factors in determining the developmental competency and thereby fertility^31^. Therefore, reduced developmental competency associate with in vitro matured oocytes can be due to reduced efficiency of the IVM system.

Based on the background literature in different species, the two-step culture system with granulosa secreted factors, including CNP before maturation, and PGE2 and AREG during IVM in the absence of FSH and LH, in order to simulate a physiological condition, similar to follicular microenvironment, for in vitro maturation of goat oocytes was proposed here.

In the first step, the percentage of oocytes arrested at the GV stage was significantly higher following CAPA treatment as compared to TCM and C.IVM groups. Several studies have reported the function of CNP on meiotic arrest in various species including goat species, Their results in goat demonstrated that CNP (100 and 150 ng/mL) can be used to delay meiotic resumption for 4 h and produced embryo by parthenogenic activation method, concluding the developmental competence of goat oocytes were enhanced^21^. Previous studies showed that in follicular microenvironment CNP binds to *NPR2* in CCs and thereby increases the concentration of cAMP in oocyte and inhibits the progress of meiosis^32^. Apparently, the bovine is an exception and in addition to CCs, DOs also have *NPR2*^18^. Similarly, previous studies reported increased meiotic arrest of ovine oocytes following the treatment with 100 and 1000 nM CNP for 24 and 12 h, respectively^10^. Another studied demonstrated that 100 nM CNP for 6 h enhanced the developmental competence of bovine treated oocytes^33,34^. We showed that CNP at concentration of 1000 nM increased the proportion of COCs remaining at the GV stage after 8 h of culture, while this treatment did not affect the GV rate in the DOs. As we mentioned, previous studies have shown that the *NPR2* is expressed and located in CCs and in collaboration with CNP (through CNP/*NPR2* pathway) participates in the maintenance of oocytes at GV stage in various species^21^ and unlike bovine *NPR2* is likely not to be expressed by goat DOs. So, CNP cannot increase GV rate in DOs in such species like swine, goat (our result) and mouse, because is only *NPR2* present on the CCs. Previous studies demonstrated that expression of CNP in mural GCs and its binding to *NPR2* in CCs increases cGMP level in CCs, which consequently leads to elevation of cAMP in oocyte. Elevated level of cAMP in oocyte stimulates the lipolysis and leads to reduction in lipid contents in bovine^35^ and porcine^36^. Our results also showed that fluorescent intensity of Nile red for lipid content decreased in goat oocytes following the treatment with CAPA. Regarding this, a previous study demonstrated that following the treatment of COCs with 100 nM CNP for 24 h, the lipid content decreased in bovine^22^. In addition, recent researches in the field of lipid metabolism have shown that CNP elevated the level of cGMP in adipocytes. Furthermore, they have shown that overexpression of CNP leads to decrease in fat weight and adipocyte hypertrophy and increases in fatty acid β-oxidation and lipolysis-related gene expression^35^.

Interestingly, in our study, we observed that following the decrease in lipid content 8 h after CNP treatment, the lipid content increased 10 h after treatment with CNP. In a previous study, it was shown that lipid droplets were newly synthesized and accumulated soon after depletion and even in the absence of fatty acids in culture medium^37^.

Following the decrease in lipid content of CNP treated COCs, we assessed the mRNA expression of genes involved in lipolysis/lipogenesis in COCs. The expression of *ATGL* and *PLIN2* in the CAPA group were significantly upregulated as compared to C.IVM group. Smirnova and colleagues demonstrated that overexpression of *ATGL* caused a reduction in the average size of lipid droplets and knockdown of *ATGL* with RNA interference led to an increase in the size of lipid droplets^38^. Therefore, reduced Nile red intensity and increased *ATGL* in CNP treated group could be related to reduced size of the lipid droplets which was unfortunately not assessed in this study. In another study, no significant difference was observed in the expression of *PLIN2* and *DGAT1* in CNP treated COCs for 24 h in bovine^22^. *PLIN2*, also known as adipose differentiation-related protein (ADRP), might be involved in the maintenance of lipid stocks necessary to support embryo development^39^. PLIN2 has been shown to be associated with lipid accumulation^22^ and acquires this effect by preventing ATGL from accessing the surface of lipid droplets^40^. PLIN2 has been identified in mouse, porcine and bovine oocytes and bovine embryos^22^. In our study *PLIN2* and *ATGL* relative expressions were concomitantly decreased in C.IVM group while the expression of both these two genes were increased in CAPA group and this likely related to maintenance of lipid accumulation as immediately after reduction of lipid content. PLIN2 needs to increase, in order to inhibit lipolysis through ATGL and may account for cyclicity in lipid content during early phase of oocyte maturation. *PLIN2* transcription is influenced by serum and cGMP levels and this effect occurs through PKG activity in bovine^41,42^. Thus, accumulation or storage of lipids can be positively influenced when cGMP levels are reduced like in C.IVM group condition, as in the presence of serum, or negatively when cGMP levels are elevated like CAPA group in our study.

PGE2 and AREG are two granulosa secreted factors which have important role in resumption of meiosis, oocyte maturation and cumulus expansion^43,44^. Thus, in the second experiment, the optimal concentration of PGE2 and AREG (1 and 600 nM, respectively) were determined. Our results indicated that CAPA-IVM-P treatment significantly improved MII and blastocyst rates, as compared with CAPA-TCM group. Our earlier study in ovine indicated that 10 µM of PGE2 for 18 h increased MII rate in ovine COCs^10^. Another study showed that no significant differences were observed in MII and cleavage rates in bovine COCs exposed to 10 µM PGE2 for 24 h but the quality of resultant embryos (in terms of grade A/B) was higher^45^. In addition, we showed that CAPA-IVM-A treatment significantly improved MII rate, blastocyst rate and TE number as compared with CAPA-TCM group. In a previous study in porcine, it was shown that exposure of COCs with 100 ng/mL AREG for 42 h increased the cleavage rate as compared with COCs that were exposed to FSH^46^. Moreover, Rouhollahi et al., showed that 300 nM of AREG resulted in a higher CEI, MII, cleavage, and blastocyst rates in ovine COCs^10^. There is a benefit of using AREG + PGE2 for stimulation of maturation over AREG or PGE2 alone, as CEI in PGE2 and AREG were 1.60 and 1.27 while CEI in PGE2 + AREG increased to 3.30. In addition, the treatment with CAPA, following treatment of COCs with a combination of PGE2 and AREG (for 18 h), resulted in improved maturation and similar blastocyst formation rate between the two procedures C.IVM and CAPA-IVM via IVF. MII rate decreased in CAPA-IVM as compared with C.IVM. No difference in CEI was observed between CAPA-IVM and C.IVM. Interestingly, TE cell number increased significantly in CAPA-IVM as compared with the C.IVM and other groups. Blastocyst rate increased significantly in CAPA-IVM as compared with TCM (24 h) and CAPA-TCM groups. In addition, no difference in cleavage rate was observed between groups. Zhang et al., also compared C.IVM group with CAPA-IVM^21^. In the last experiment, the treated COCs were subjected to SCNT procedure and our results showed no significant difference in the developmental rate between all experimental groups, but the ICM number, TCN and ICM/TE ratio in CAPA-IVM were higher as compared to C.IVM. In this regard, previous studies have shown that even a small increase in ICM count has a special role in increasing mouse fetal function^47,48^. Another study showed that adding EREG and AREG to the maturation culture medium in mice can increase the number of ICM the blastocysts^49^.

In addition, we assessed the quality of SCNT embryos through assessing the mRNA expression of pluripotency genes including *NANOG*, *SOX2*, and *OCT4*. In mammals, transcription of these three genes are regulated by a loop signaling in a steady-state, despite differences in various species. Therefore, lowering one factor will lead to the up-regulation of one or two other factors^50^. A study in goat species has shown that embryos showing a decrease in *NANOG* expression also show a decrease in the number of TE cells. *NANOG* is likely to be required for TE cell proliferation^50^. We showed that *NANOG* expression was increased in the embryos of treatment group as compared to C.IVM group.

Our data also revealed a higher but not significant re-expansion rate of blastocysts in CAPA-IVM group as compared with C.IVM group. The re-expansion rate in CAPA-IVM group was significantly higher than TCM (24 h) group.

In the present study, to evaluate post-implantation growth, embryo transfer was performed after vitrification-thawing procedure in C.IVM and CAPA-IVM group. Despite, the increase in pregnancy rate and decrease in pregnancy loss in CAPA-IVM as compared to C.IVM group but the difference were not significant. Also, we showed higher live births rate, and SCNT efficiency in CAPA-IVM, although these values were no significantly different which is likely related to small sample size. The results of in vivo studies in goats show the need to improve SCNT method^51^.

Another study showed a significantly higher live birth rate after transfer of frozen versus fresh blastocysts (day 5 embryo) in women with a regular menstrual cycle undergoing IVF with capacitation IVM^52^. Human studies have shown that MII rate and embryonic development is improved in immature oocytes exposed to factors secreted by GCs as compared with immature oocytes in absence of granulosa secreted factors^53–56^. In this way, supplementation of maturation medium with granulosa secreted factors during IVM led to improvement of IVP.

To improve the CAPA-IVM system, according to Richani et al., it is suggested to regulate lipid metabolism by L-carnitine during in vitro maturation^57^. In general, studies are moving towards the use of new IVM system namely “the CAPA-IVM”. It should be noted that in the present study, for the first time, both AREG and PGE2 factors were used in a CAPA -IVM in goat species, and also for the first time, the effects CAPA-IVM method on pre- and post-implantation development of SCNT were assessed. In recent advances in human IVM, CNP in the pre-IVM stage and AREG are used^28,54,56^. In addition, the CAPA-IVM improves the quality of IVM oocytes in mice^58^.

In SCNT procedure, in contrast to IVF, due to high manipulation, a good oocyte maturation could have a more profound effect in the final result. In addition, improved application of SCNT in therapeutic cloning, producing animal models for human diseases, conservation of endangered animals and production of a large number of embryos from genetically elite animals, especially in animal breeding program, has made SCNT a valuable tool in biotechnology and therefore, improve efficiency of SCNT may have paramount importance. Therefore, this study highlight, the importance of improving IVM not only for production of embryo through IVF but also for future biotechnology through SCNT.

## Conclusion

In physiological condition, the oocyte become mature in close communication with mural and GCs and therefore, factors secreted by these cells play a paramount role in oocyte maturation and subsequent developmental competence. According to this study, substituting LH and FSH with the granulosa secreted factors for temporary meiotic arrest and subsequently stimulating maturation in hope of increasing the quality of embryos and subsequent developmental competency may have profound effect in assisted reproduction and future biotechnology.

## Material and methods

### Media and reagents

All chemicals and media were obtained from Sigma Aldrich Chemical Co. (St. Louis, MO) and Gibco (Grand Island, NY), respectively, unless stated. The Institutional Review Board and Institutional Ethical Committee of the Royan Institute approved all animal care protocols and the proposal (90000001). In addition, all methods used in this study were handled according to the guidelines and regulations provided by the Institutional Review Board and Institutional Ethical Committee of the Royan Institute.

### Ethics and animals

All procedures were approved by the Institutional Review Board of Royan Institute, Tehran, Iran. All animal experiments were conducted in compliance with the ethical guidelines established by the Institutional Ethics Committee of Royan Institute. Also, the manuscript follows the recommendations in the ARRIVE guidelines.

### In vitro maturation

Goat ovaries obtained from slaughterhouse were transported to the laboratory at 17–19 °C. Ovaries were maintained in normal saline at 15–18 °C for 12 h until aspiration^59,60^. Follicles were aspirates with a 22-gauge needle attached to a vacuum pump into a conical tube. COCs with more than three layers of CCs and homogenous cytoplasm were selected and washed in HEPES tissue culture medium (HTCM) droplets which were supplemented with 1 mg/mL polyvinyl alcohol (PVA) and 4 mg/mL bovine serum albumin (BSA). Finally, COCs were cultured in a 500 µl IVM medium in 4-well dish containing TCM + 1 mg/mL PVA + 8 mg/mL BSA in each group, according to experimental design without mineral oil at 38.5 °C, 5% CO2 and humidified atmosphere. The C.IVM consisted in TCM + LH (10 µg/ml) + FSH (10 µg/ml) + IGF (100 ng/ml) + EGF (10 ng/ml) + Cysteamine (0.1 mM) + FBS (15%) + E2 (1 µg/ml).

### Nuclear status

CCs were removed from COCs by vortexing with 300 IU/mL hyaluronidase. The DOs were washed 3 times in phosphate-buffered saline (PBS^-^) + PVA. Afterward the DOs were fixed for 20 min in 4% paraformaldehyde (PFA). Then, they were stained with Hoechst 33,342 (5 µg/mL) for 7 min. The stained DOs were transferred to a glass slide containing 5 µl mounting fluid and covered with a cover glass^10^. The images of the stained oocytes were captured and assessed by a high-resolution digital camera (DP-72 Olympus) using DP2-BSW software. The percentages of the oocytes at the GV and MII stages in each experimental group were determined.

### Cumulus expansion assessment

After 24 h of maturation, CEI of matured COCs in various groups was scored on a zero to four scale. Score zero no expansion, score one no CCs expansion but cells seem as spherical; scale two only the outer most layers of CCs have expanded; scale three all cell layers except to corona radiata have expanded and score four expansion occurring in all cell layers^61^.

### Lipid content

In order to assess the lipid content of matured oocytes in various experimental groups, after washing the DOs in PBS + PVP solution and fixation with 4% PFA, the DOs were stained with 1 µg/mL Nile red (Sigma, N1142) for 30 min. After mounting the oocytes on glass slides, the fluorescence signal was immediately detected under an epifluorescence microscope (Olympus BX51) with a high resolution digital camera (DP-72 Olympus) using DP2-BSW software^62^. The fluorescent intensity was measured using Image J software (ij142-jdk6-setup.exe, National Institute of Mental Health, Bethesda, Maryland, USA). The lipid content is presented as the mean of the fluorescence intensity ± SEM^36^.

### Relative gene expression

COCs with three layers of CCs and approximately same CCs number were transferred and stored in RLT buffer at − 70 °C until RNA extraction. Total RNA has been extracted from a pool of 10 COCs and a pool of 5 expanded blastocysts (including grade I blastocysts that exhibit uniform blastomeres and no fragmentation). The RNA was extracted using RNAeasy Micro Kit (Qiagen, Germany) according to the manufacturer’s instructions. DNA digestion was followed by using DNase I treatment. The RNA was reverse transcribed into complementary DNA (cDNA) using the Biotechrabbit kit (USA).

Relative gene expression of target genes in COCs (including *ATGL*, *PLIN2*, and *DGAT1*), and embryos (including *SOX2*, *NANOG*, and *OCT4*) (Table 3) were performed using SYBRGreen by real-time cycler step-one PLUS ABI (ABI, USA). Values of relative expression of target genes in this study were normalized to geomean of reference genes *β-actin* and *GAPDH* and 2^−∆∆CT^ was presented.

**Table 3:** Primer design.

Symbol Gene	Forward Primer	Reverse Primer	Annealing Temp. (°C)	Length
*ATGL*	GTATTACTCCTGAGAACTT	GTGGCGTTTATTACATCT	52	113
*PLIN2*	GATTGAACTTGCCAGGAA	CAGCCAGGACAGATAGAG	52	82
*DGAT1*	GCATCCTGAATTGGTGTG	CCGTACTTGATGAGGTTCT	52	73
*SOX2*	ATGGGCTCGGTGGTGA	CTCTGGTAGTGCTGGGA	55	82
*OCT4*	GGAAAGGTGTTCAGCCA	ATTCTCGTTGTTGTCAGC	57	123
*NANOG*	GATTCTTCCACAAGCCCT	TCATTGAGCACACACAGC	53	137
*β-ACTIN*	CCATCGGCAATGAGCGGT	CGTGTTGGCGTAGAGGTC	57	146
*GAPDH*	GGCATCGTGGAGGGACTT	GGAGGCCATGTGGACCA	55	146

### In vitro fertilization

To verify the ability of the matured oocytes to develop into blastocyst stage, oocytes were subjected to IVF and IVC procedure. Matured COCs were washed in fertilization medium (NaCl 114 mM, KCL 3.15 mM, NaH_2_PO_4_ 0.39 mM, Na-Lactate 13.3 mM, CaCL_2_ 2 mM, MgCL_2_ 0.5 mM, Na-Pyruvate 0.2 mM, penicillin 50 IU/ ml, Streptomycin50 µg/ml, NaHCO_3_ 25 mM, Heparin 10 µg/ml, BSA 6 mg/ml). After that, the frozen goat sperm was processed by swim-down method. Then, insemination was performed with 1 × 10^6^/mL sperm added to each 50 µl drop of fertilization medium containing 10 oocytes for 18 h under 5% CO_2_, humidified atmosphere at 38.5 °C and covered with mineral oil (Sage, ART-4008-5p)^10^.

### Somatic cell nuclear transfer (SCNT)

The SCNT procedure was performed similar to the method described previously^63^. In brief, CCs were first removed via vortexing of COCs in HTCM + 1 mg/mL PVA + 4 mg/mL BSA containing 300 IU/mL hyaluronidase. Then the zona pellucida was removed by exposing the DOs to H-TCM + %10 FBS + 2.5 mg/mL protease for 60 s. After that zona-free oocytes were incubated for 20 min in H-TCM + 20% FBS. In this study, oocyte enucleation was performed using the manual method as described previously^63^. During this procedure, oocytes were first treated with 0.4 µg/mL demecolcine (H199 with 15% serum, 10 mg/mL BSA, 3 mg/mL PVA, and 0.4 μg/mL demecolcine) for 20 min at 38.5 °C. By treating the oocytes with demecolcine, a protrusion becomes apparent on the surface of cell membrane of oocytes which contains the nucleus of the oocyte. Enucleation was performed through manual oocyte enucleation using a fine pulled Pasteur pipette. The cytoplasmic protrusion was removed using a hand-held manual oocyte enucleation pipette. For nuclear replacement, the enucleated oocytes were transferred to droplets of H-TCM, supplemented with 10 mg/ml phytohemagglutinin, and well-rounded fibroblast cells were attached to the membrane of the enucleated oocytes. Next, the couplets in the fusion buffer, were electro-fused using a sinusoidal electric current (7 V/cm) for 10 s, followed by two direct currents (1.75 kV/cm for 80 μs and a time delay of 1 s). After 30-min, oocyte activation was induced by the incubation of reconstructed oocytes with 5 μM Ionomycin for 1 min, followed by incubation for 2 h with 2 mM 6-dimethylaminopurine (6-DAMP). The activated reconstructed oocytes were then cultured inside the wells, which contained SOF^+^^63,64^ at 38.5 °C in a humidified atmosphere with 5% CO_2_ and 5% O_2_ for 7 days under mineral oil. The embryos were then evaluated for cleavage and blastocyst rate on day 3 and 7, respectively.

### Differential staining

In order to evaluated the quality of blastocyst, differential staining were used^65^. In this protocol inner cell mass (ICM) and trophectoderm (TE) cells appear in blue and pink under UV light, respectively. To achieve this goal day 7 hatched IVF and SCNT derived blastocysts were washed in HTCM and 5 mg/mL BSA. Then, the blastocysts were exposed to 0.5% Triton X-100 and subsequently to 30 mg/mL propidium iodide for 30 s and 1 min, respectively. Consequently, embryos were stained and fixed in cold solution (4 °C) containing ethanol and 10 mg/ml Hoechst for 15 min. Finally, the blastocysts were mounted in mounting solution and examined by using a fluorescent microscope.

### Vitrification-thawing procedure

For cryopreservation of goat embryos by vitrification method, day 7 blastocysts were washed in basic medium (BM) (PBS^-^ + 20% FBS) for 1 min, equilibrium solution (BM + %7.5 DMSO + %7.5 Ethylene glycol (EG)) for 5 min and vitrification solution (BM + %15 DMSO + %15 EG + 0.5 M sucrose) for 35 s. The embryos were then immediately placed on the Cryotop (KITZATO/Cryotop) in minimal medium and then directly transferred to liquid nitrogen.

For thawing, the tip of the Cryotop holding the vitrified embryo was placed in a drop of thawing solution 1 (BM + 1 M Sucrose) for 1 min until the embryos are separated from the Cryotop, then these embryos were washed in thawing solution 2 (BM + 0.5 M Sucrose) for 3 min and BM medium for 5 min and finally transferred to SOF^+^ medium at 38.5 °C, 5% O_2_% and 5% CO_2_ and maximum humidity until embryo re-expansion.

### Embryo transfer

Bakhtiari recipient goats with at least one parturition were checked by an expert veterinarian for normal appearance and health, and screened for contagious diseases including Johne’s disease and brucellosis. The selected goats were 2–3 years old and had a mean weight of 35 kg. SCNT thawed grade I and II blastocysts were transferred to recipient on day after thawing. They were synchronized with insertion of progesterone sponge (40 mg fluorogestone acetate, Intervet™) which was considered as day 0. Subsequently, injection of 500 IU PMSG (Pregnant Mare’s Serum Gonadotropin)/goat, 250 μg/goat of prostaglandin (estroPLAN®, ustralia) and 1000 IU/goat of hCG (human chorionic gonadotropin) were carried out on day 5, 7 and 9, respectively^59^.

On the day of embryo transfer, the embryos were removed from their culture medium and washed in H-TCM + 10% FBS droplets. Using a pipette and H-TCM + 10% FBS medium, embryos were pulled into special catheter for embryo transfer. The catheter was then transferred to farm using special flasks. Embryo transfer was also performed by laparoscopy with subcutaneous injection of lidocaine (0.1 g/animal, Kwangmyung Pharm, Korea) for local anesthesia^66^. During the laparoscopic operation, a small umbilical incision (about 1 cm) was inserted into the abdomen. After observing the uterus and fallopian tube, 2 grad I and II blastocyst were transferred to the uterine horn. The pregnancy status of the recipient animals was assessed using rectal ultrasound on day 28–38 and abdominal ultrasound on days 83–113 after embryo transfer. All animals were allowed to give birth naturally on day 150 ± 5^59^.

### Statistical analysis

Normality of data and equality of variances were checked using Kolmogorov–Smirnov and Levene tests, respectively. The statistical analysis was performed using SPSS18.0 statistical software (IBM Corporation, Somers, NY). Values are expressed as mean ± standard error of the mean, and level of significance was at *P* ≤ 0.05. CEI was analyzed using a nonparametric Kruskal–Wallis test. Other data in the current study were examined using One-way ANOVA followed by Tukey’s post-hoc and two independent sample t-Test. The figures were drawn with GraphPad Prism version 9.2.0 software.

### Experimental design

In the first experiment (Fig. 1A), best time and concentration of CNP was determined to retain oocytes in GV stage and reduced lipid content. Firstly, COCs were exposed to different concentrations of CNP (10, 100 and 1000 nM) for different duration of culture (0, 6, 8, 10, 12 and 24 h) to determine the optimal concentration and duration for the highest rate of oocyte in the GV stage. CNP work through *NPR2* in CCs and not oocyte, to verify this point in goat, DOs were also exposed to 1000 nM CNP for 8 h and percentage of arrest in GV stage were compared to TCM (Fig. 1A). Then, effect of CNP (10, 100 and 1000 nM) for different duration of culture (0, 6, 8, 10, 12 and 24 h) on the lipid content of oocytes was evaluated (Fig. 1A). Finally, relative genes expression related with lipid metabolism (*ATGL*, *PLIN2* and *DGAT1*) was assessed in COCs (Fig. 1A). This was a pre-maturation culture period named as capacitation or CAPA.

The second experiment was designed to determine the optimal concentrations of PGE2 and AREG for stimulating maturation for 18 h. Control group here was CAPA-TCM. In treatments group, immature COCs were initially exposed to CAPA and then exposed to different concentrations of PGE2 (0.1, 1, and 10 nM) and/or AREG (100, 300 and 600 nM) for 18 h. MII rate, CEI, developmental rate and blastocyst cell number were assessed and comparing to CAPA-TCM (Fig. 1B). The best concentration group was named CAPA-IVM-P and CAPA-IVM-A (Fig. 1B). After determined best condition of CNP, PGE2 and AREG, COCs were exposed to CAPA then exposed to optimal concentrations of PGE2 and AREG (CAPA-IVM) and percentage of MII, CEI and developmental rate as well as blastocyst cell number were assessed through IVF procedure. The final selected group was named CAPA-IVM (Fig. 1C).

After determining the CAPA-IVM condition, the third experiment was in vitro and in vivo development of SCNT embryos in CAPA-IVM. The SCNT blastocysts were assessed for differential staining, ICM/TE ratio, the relative expression of pluripotency related genes (*NANOG*, *SOX2* and *OCT.4*) (Fig. 1D). Additionally, the blastocysts were vitrified-thawing and their re-expansion rate were determined (Fig. 1D). Finally, SCNT embryos were transferred to goat recipient. Post implantation developmental competence of SCNT were also compared between the CAPA-IVM and C.IVM (Fig. 1D). Therefore, the experimental groups were defined as below:

$$\begin{aligned} & {\text{TCM}}:\left[ {{\text{TCM }} + {\text{ BSA }} + {\text{ PVA }}\left( {{\text{24h}}} \right)} \right], \\ & {\text{CAPA}} - {\text{TCM}}:\left[ {{\text{CNP }}\left( {{1}000{\text{ nM}},{\text{8h}}} \right) \to {\text{TCM }}\left( {{\text{18h}}} \right)} \right], \\ & {\text{C}}.{\text{IVM}}:\left[ {{\text{TCM }} + {\text{ LH }} + {\text{ FSH }} + {\text{ IGF }} + {\text{ EGF }} + {\text{ Cysteamine }} + {\text{ FBS }} + {\text{ E2 }}\left( {{\text{24h}}} \right)} \right], \\ & {\text{CAPA}} - {\text{IVM}} - {\text{P}}:\left[ {{\text{CNP }}\left( {{1}000{\text{ nM}},{\text{8h}}} \right) \to {\text{PGE2 }}\left( {{\text{1nM}}} \right) \, \left( {{\text{18h}}} \right)} \right], \\ & {\text{CAPA}} - {\text{IVM}} - {\text{A}}:\left[ {{\text{CNP }}\left( {{1}000{\text{ nM}},{\text{8h}}} \right) \to {\text{AREG }}\left( {{6}00{\text{nM}}} \right) \, \left( {{\text{18h}}} \right)} \right] \\ & {\text{CAPA}} - {\text{IVM}}:\left[ {{\text{CNP }}\left( {{1}000{\text{ nM}},{\text{8h}}} \right) \to {\text{PGE2 }}\left( {{\text{1nM}}} \right) \, + {\text{ AREG }}\left( {{6}00{\text{nM}}} \right) \, \left( {{\text{18h}}} \right)} \right]. \\ \end{aligned}$$

## Data Availability

Data supporting the manuscript’s findings can be found in the manuscript.
